# Cystic Change in Pleomorphic Adenoma: A Rare Entity

**DOI:** 10.7759/cureus.81683

**Published:** 2025-04-04

**Authors:** Vattikuti Satya Veni, Atla Bhagyalakshmi, Sri Keerthika, Roohi Firdous

**Affiliations:** 1 Pathology, NRI Institute of Medical Sciences, Visakhapatnam, IND

**Keywords:** cystic change, encapsulated lesion, epithelial component, parotid, pleomorphic adenoma

## Abstract

Pleomorphic adenomas most frequently affect the parotid gland, primarily the superficial lobe. The cystic change, which can resemble squamous cell carcinoma, mucoepidermoid carcinoma, mucocele, or carcinoma ex pleomorphic adenoma, presents a diagnostic challenge in pleomorphic adenomas. Here, we report a unique and intriguing case of cystic pleomorphic adenoma in a 41-year-old man.

## Introduction

Pleomorphic adenoma (PA) is the most common salivary gland tumor, affecting the parotid gland [1]. This benign tumor is characterized histologically by a mix of epithelial and mesenchymal elements, giving rise to its alternate name, "benign mixed tumor" [2]. Typically presenting as a firm, slow-growing, well-circumscribed mass, PA can occasionally exhibit atypical features such as cystic degeneration.

Cystic changes, though rare, are generally secondary phenomena caused by necrosis, hemorrhage, or stromal hyalinization and can complicate diagnostic evaluations through fine-needle aspiration cytology (FNAC) [3]. These atypical presentations, while uncommon, are clinically significant, as untreated PAs carry the risk of malignant transformation [4]. This report highlights a rare case of cystic change in PA, emphasizing the diagnostic and management challenges posed by this unusual entity.

## Case presentation

A 41-year-old man presented with a painless swelling in the right parotid region that had persisted for three months. No other significant history was reported. On examination, a 4 × 3 cm firm mass was identified in the right parotid area. Ultrasonography revealed a cystic, well-encapsulated lesion with minimal vascularity and an eccentric solid component, suggestive of Warthin’s tumor. FNAC was done, and based on the microscopic features showing small cohesive sheets of ductal epithelial cells and granular debris with sparse lymphocytes, a provisional diagnosis of Warthin's tumour was made. Due to quality concerns, microscopic pictures are not available. Subsequently, a superficial parotidectomy was performed.

**Figure 1:**
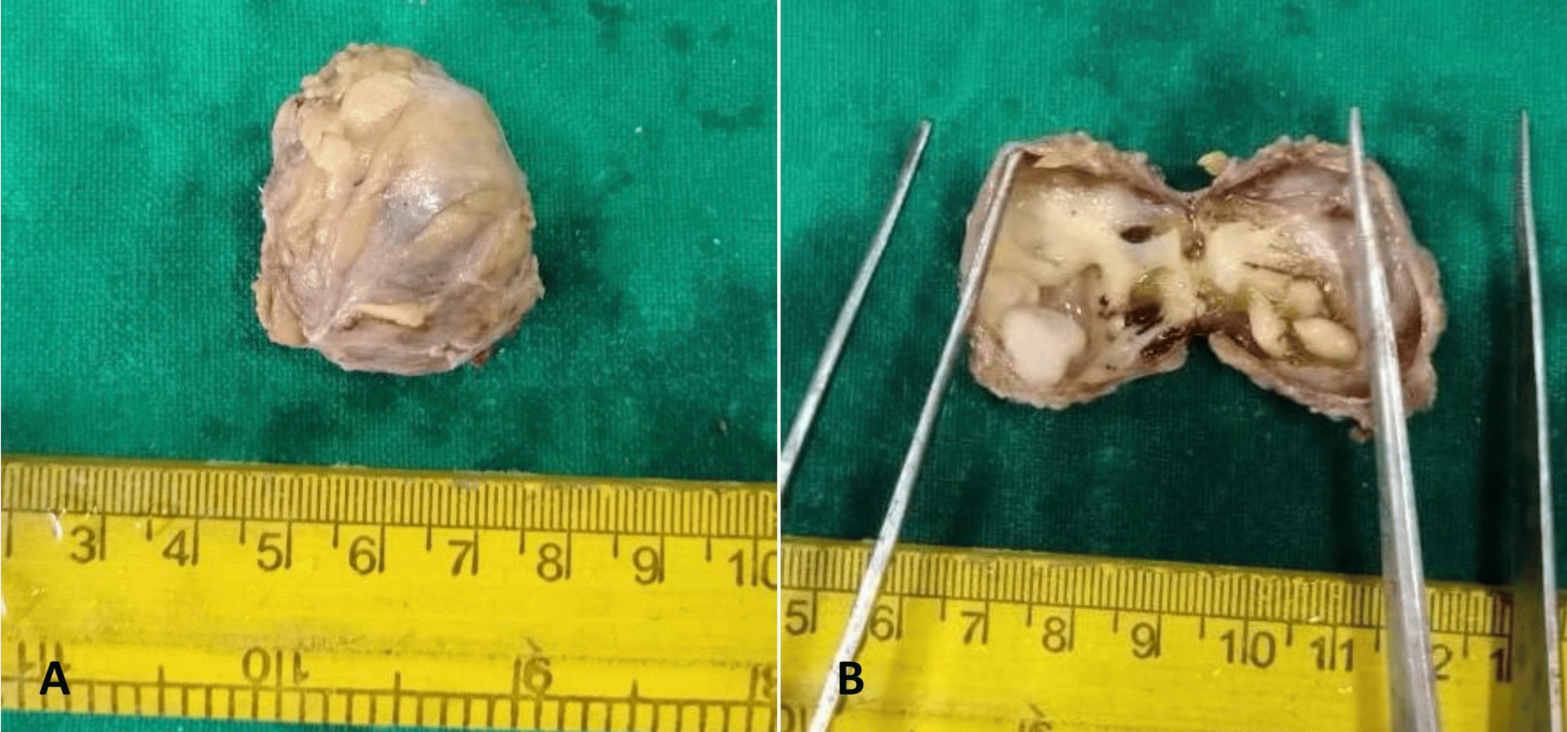
(A) Gross image showing a gray-brown irregular nodular mass. (B) Cut section showing a single cystic cavity having a smooth surface filled with gelatinous material

Gross examination of the excised specimen revealed a nodular mass measuring 3 × 2.5 × 2 cm. The external surface was irregular (Figure 1A). On the cut section, a large cystic area measuring 2.7 × 2 cm was noted, accompanied by a peripheral gray-white firm area with a wall thickness of 0.1 cm (Figure 1B). Additionally, a single small lymph node measuring 1.5 × 1.5 cm and a separately sent bit of soft tissue mass, probably of parotid origin, measuring 1.8 × 1.5 cm were received.

**Figure 2:**
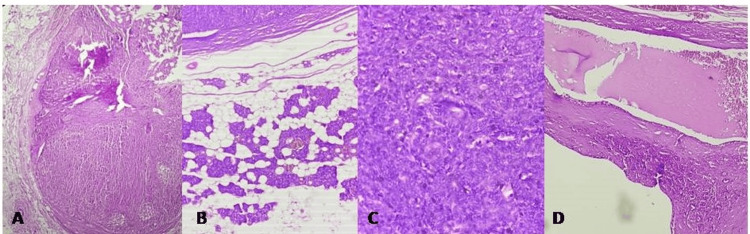
Photomicrographs showing a cystic well-encapsulated lesion with solid area composed predominantly of epithelial component (A, H&E, 4×); normal salivary gland component showing acini (B, H&E, 4×); epithelial component predominantly arranged in sheets, ducts, cords, and acini (C, H&E, 10×); cystic cavity lined by columnar epithelium with eosinophilic secretions (D, H&E, 10×)

Microscopic analysis showed a well-encapsulated lesion with solid regions primarily composed of an epithelial (ductal) component arranged in cords, ducts, sheets, and acini. The cystic spaces contained eosinophilic secretions and an epithelial component forming the inner layer of the cyst with adjacent normal salivary gland tissue. The photomicrographs of the lymph node showed reactive changes. The solid area was composed predominantly of epithelial components (Figure 2A). Normal salivary gland acini were also visible (Figure 2B). The epithelial component was arranged in sheets, ducts, cords, and acini (Figure 2C). A diagnosis of PA with cystic change was made.

## Discussion

PAs are the most common salivary gland neoplasms, accounting for approximately 64% of cases [5]. These mixed tumors are characterized by both mesenchymal and epithelial components [6]. Although PA is notable for its histological diversity, this complexity can occasionally pose challenges in accurate interpretation [7,8]. Cystic changes in PAs can present a diagnostic dilemma, as they may mimic conditions such as mucocele, squamous cell carcinoma, mucoepidermoid carcinoma (MEC), or carcinoma ex PA [9].

PA is marked by diverse morphological features, architectural patterns, and cellular composition [9]. It displays epithelial and chondromyxoid stroma, varying histological patterns within tumor sections, metaplastic changes, and cystic areas [7]. FNAC plays a pivotal role in evaluating salivary gland tumors. It helps avoid unnecessary surgery in some cases and guides preoperative planning, determining whether conservative or extensive surgical intervention is required [5]. Aspirated material from PAs typically appears as thick, mucoid droplets. The cytological samples predominantly include extracellular matrix and stroma, with varying amounts of myoepithelial and ductal cells depending on the tumor’s cellularity [10].

While FNAC is typically a straightforward method for diagnosing PA, cystic variants present unique diagnostic challenges. Diagnosing common salivary gland neoplasms such as PA via FNAC is generally easy; however, factors such as altered differentiation, degeneration, and metaplastic alterations like cystic change can complicate the process [5]. Cystic PAs with metaplastic epithelial alterations require a careful and systematic approach to both cytological and histopathological evaluation, as highlighted by Siddaraju et al. [8]. Due to significant variability in the proportions of tumor components, these lesions may be misinterpreted as low-grade carcinomas, monomorphic adenomas, metastases, or even malignant lymphomas or plasma cell proliferations because of the plasmacytoid appearance of myoepithelial cells [10].

The histology of the current case revealed localized secondary changes and alterations. Diagnosing such changes can be challenging, as squamous metaplasia in benign salivary gland tumors may mimic malignant neoplasms such as MEC or squamous cell carcinoma. This diagnostic difficulty may arise from the limited mesenchymal component in PAs and the nuclear atypia of squamous cells.

Low-grade MEC is often challenging to exclude in cytologic preparations due to its bland appearance and the difficulty in identifying mucin-containing cells. In contrast, the diagnosis of PA is facilitated by the presence of the characteristic metachromatic fibrillar chondromyxoid stroma typically seen in PAs. Although cytologic diagnosis is helpful, resection is recommended because additional cellular components may complicate interpretation.

To avoid misdiagnosing PA with squamous metaplasia as MEC on cytology, it is essential to carefully examine smears for fragments of chondromyxoid stroma. When metaplastic changes such as squamous, mucinous, or sebaceous metaplasia are present, FNAC may erroneously suggest malignancy, particularly MEC, especially if chondromyxoid stroma is absent [6].

In cases where subsequent parotidectomy reveals an encapsulated cystic PA with mixed appendage differentiation, the diagnosis can be confirmed histologically. This is crucial when low-grade MEC is part of the differential diagnosis. Diagnostic challenges often arise from sparse cellularity, limited sampling, and observer unfamiliarity with the tumor’s variable patterns. Without chondromyxoid stroma, features such as basaloid cells, hyaline globules, and squamous metaplasia can be misinterpreted as carcinoma.

A review of the literature reveals that cases of PA with cystic transformation are surprisingly rare [7,9]. Cyst formation in PAs can result from hemorrhagic infarction, squamous metaplasia of epithelial cells, or tissue secretions from tumor cells or salivary glands, which enlarge duct-like structures. Squamous metaplasia is an uncommon microscopic finding in benign salivary gland tumors, often associated with necrosis and regeneration of salivary gland tissue following ischemia or minor trauma.

In some cases, areas of the lining epithelium may appear flattened or denuded. Cellular changes resembling malignancy may accompany cystic changes in PA of minor salivary glands; however, no cellular atypia or mitosis was observed in the present case [9]. Extensive squamous metaplasia with cystic transformation, especially in the absence of stroma, can lead to limited sampling by FNAC or incisional biopsy, increasing the risk of misdiagnosis. Benign conditions such as keratocystoma or malignancies like MEC or squamous cell carcinoma may be mistaken for PA under these circumstances. Reports in the literature have documented adnexal differentiation, characterized by florid squamous metaplasia and keratin-filled cysts.

To avoid misdiagnosing PA with squamous metaplasia as malignancy, it is crucial to carefully examine the sample for the presence of chondromyxoid stroma, a hallmark of PA.

The primary management of PA includes surgical excision along with the adjacent tissue to ensure complete removal and prevent recurrence.

## Conclusions

Cystic PA is a rare and diagnostically challenging variant of the most common salivary gland tumor. Its resemblance to Warthin's tumor, MEC, or squamous cell carcinoma highlights the need for meticulous histopathological evaluation. Fine-needle aspiration cytology is valuable but may be inconclusive in cystic cases, necessitating excision for definitive diagnosis. Surgical resection remains the treatment of choice, addressing both the risk of recurrence and potential malignant transformation. Awareness of this uncommon presentation is essential for accurate diagnosis and optimal management.
